# Temporal Characteristics of the Oropharyngeal and Nasal Microbiota Structure in Crewmembers Stayed 180 Days in the Controlled Ecological Life Support System

**DOI:** 10.3389/fmicb.2020.617696

**Published:** 2021-02-03

**Authors:** Yanwu Chen, Chong Xu, Chongfa Zhong, Zhitang Lyu, Junlian Liu, Zhanghuang Chen, Huanhuan Dun, Bingmu Xin, Qiong Xie

**Affiliations:** ^1^Space Science and Technology Institute (Shenzhen), Shenzhen, China; ^2^China Astronaut Research and Training Center, Beijing, China; ^3^Key Laboratory of Microbial Diversity Research and Application of Hebei Province, College of Life Science, Baoding, China

**Keywords:** controlled ecological life support system, microbial community, space flight, oropharynx, nasal cavity, microbiota

## Abstract

Confined experiments are carried out to simulate the closed environment of space capsule on the ground. The Chinese Controlled Ecological Life Support System (CELSS) is designed including a closed-loop system supporting 4 healthy volunteers surviving for 180 days, and we aim to reveal the temporal characteristics of the oropharyngeal and nasal microbiota structure in crewmembers stayed 180 days in the CELSS, so as to accumulate the information about microbiota balance associated with respiratory health for estimating health risk in future spaceflight. We investigated the distribution of microorganisms and their dynamic characteristics in the nasal cavity and oropharynx of occupants with prolonged confinement. Based on the 16S rDNA v3–v4 regions using Illumina high-throughput sequencing technology, the oropharyngeal and nasal microbiota were monitored at eight time points during confinement. There were significant differences between oropharyngeal and nasal microbiota, and there were also individual differences among the same site of different volunteers. Analysis on the structure of the microbiota showed that, in the phylum taxon, the nasal bacteria mainly belonged to Actinobacteria, Firmicutes, Proteobacteria, Bacteroidetes, etc. In addition to the above phyla, in oropharyngeal bacteria Fusobacterial accounted for a relatively high proportion. In the genus taxon, the nasal and oropharyngeal bacteria were independent. *Corynebacterium* and *Staphylococcus* were dominant in nasal cavity, and *Corynebacterium, Streptococcus*, and *Neisseria* were dominant in oropharynx. With the extension of the confinement time, the abundance of *Staphylococcus* in the nasal cavity and *Neisseria* in the oropharynx increased, and the index Chao fluctuated greatly from 30 to 90 days after the volunteers entered the CELSS.

**Conclusion:** The structure and diversity of the nasal and oropharyngeal microbiota changed in the CELSS, and there was the phenomenon of migration between occupants, suggesting that the microbiota structure and health of the respiratory tract could be affected by living in a closed environment for a long time.

## Introduction

In the human respiratory tract, there is a complex microbiota and microecological balance, and the disruption of the balance at specific sites may lead to the overgrowth of pathogens and the increased susceptibility to infection. Infection is generally associated with altered microbial diversity and microbiota structure and that had been reported in a variety of respiratory diseases, including upper respiratory tract infection accompanied by acute otitis media (AOM) (Chonmaitree et al., 2017), pharyngitis (González-Andrade et al., 2017), asthma (Katsoulis et al., 2019), and pneumonia (Morinaga et al., 2019). Nasal cavity and oropharynx locate the entrance of the upper respiratory tract, which serve as the physical barrier to the invasion of the pathogens and also important habitats colonized by a large number of conditional pathogen. Clinical pathogenic bacterium, such as *Staphylococcus aureus, Streptococcus pneumoniae, Haemophilus influenzae*, and *Moraxella catarrhalis* are generally colonized in the nasal cavity. Thus, it is also a main position of the viral infection (de Steenhuijsen Piters et al., 2015; Brugger et al., 2016; Bomar et al., 2018). The oropharynx is an important site for the colonization of pathogenic bacteria. Metagenomic sequencing has proved that the pharynx of a healthy adult is colonized by pathogenic bacteria such as *Streptococcus, Haemophilus* and *Neisseria* (Segata et al., 2012; Ver Heul et al., 2019). Normally, these pathogenic microorganisms live in the host as part of the microecosystem, occupying an ecological niche and even resisting the infection of other exogenous pathogenic bacteria. However, in a few cases, they cause respiratory diseases, which need to be triggered by various exogenous or endogenous stimuli (Bogaert et al., 2004). Dynamic surveillance of oropharyngeal and nasal microbiota structure may be crucial in predicting inflammatory lung disease and guiding medical treatment (Lee et al., 2019).

During space flight, the astronauts will have to live in the spacecraft for a long time. Factors like confined living environment, lifestyle changes, stress, and biological rhythm changes may have a significant impact on the physiological environment and the body's immune function. On the other hand, the changes of environmental stress factors, such as oxygen, pH, humidity, and nutrient, may cause the dynamic response of symbiotic bacteria. The uncontrollable increase of pathogenic bacteria in the bacterial community may lead to infection. The experience of the manned space flight of the United States and Russia has proved that with the extension of flight time, the accumulation of microorganisms in the cockpit became more and more serious, and the increase of pathogens in the living environment was more likely to cause infection and allergy and affect human health. Dynamic studies of microbes in confined spaces have shown that harmful bacteria accumulated in the environment with prolonged confined time and it weakened the immune system of humans exposed to stress and extreme environmental conditions during space flight, leading to increased susceptibility. In aerospace health events, upper respiratory symptoms (0.97 per flight year) and other (non-respiratory) infectious events are among the most prevalent, second only to rashes (1.12 per flight year) (Crucian et al., 2016a).

Detection and analysis of on-orbit microorganisms can provide a comprehensive understanding of the microbial composition and changes in pathogenic microorganisms on the space station, which is conducive to the prevention of infectious diseases (Ichijo et al., 2016; Blachowicz et al., 2017; Lang et al., 2017).

In this study, the CELSS was used to simulate space environment on the ground, and four crewmembers were confined for 180 days in the experiment. With Illumina 16S rDNA V3–V4 high-throughput sequencing technologies, the systemic research of human source bacteria microbiota was carried out, in order to research microbial characteristics of the upper respiratory tract of the crew living in a closed environment for a long time. The risk of suffering from respiratory disease for humans living in an airtight environment was assessed, and we aimed to provide reference basis for infection control on orbit.

## Materials and Methods

### Volunteers

The crewmembers consisted of 3 males and 1 female (age 34.2 ± 6.6 years, weight 64.5 ± 6.1 kg, height 169.3 ± 5.1 cm) that were selected from 2,110 volunteers through qualification examination and physical and psychological examinations. In this study, infectious disease including chronic pharyngitis, asthma, and pneumonia would be causes for rejection. Crewmembers were adequately trained to perform the related test tasks before the experiment.

The research program was approved by the Ethical Committee of the ACC (China Astronaut Research and Training Center, Beijing, China) and complied with all the guidelines in the Declaration of Helsinki. Each volunteer was informed of the content and schedule of the study, and the informed consent form was signed. Participants can withdraw from the study at any time.

### Research Design

The CELSS platform consists of six interconnected modules and eight compartments, including two crew pods, four greenhouses, a resource pod, and a recovery and purification system to treat waste (feces, urine, plant residues, wastewater, exhaust gas) and produce CO_2_ for plants, as well as a life support pod for storing and processing food. The crew cabin consists of a single bedroom, a working area, a medical monitoring area, a cafeteria, and a gym. The life support system is controlled by an automatic feedback network.

The four crewmembers follow a strict diet and working schedule. Meanwhile, they control water treatment units, plant culture, garbage disposal, life support, and air control systems, and they perform cleaning and maintenance tasks (Yuan et al., 2019). In addition, they actively engage in scientific experiments in which they are the subjects of many psychological and physical tests. The project described in this paper is one of these experiments, named “Confined Habitat and Microbial Ecology of Human Health” experiment, which aims to obtain detailed data on microbiota changes in human from confined environments. Nasal and oropharyngeal samples were taken from four crew members during the experiment which lasted 180 days. Monthly sampling was conducted on the same day or one day before the delivery so that all the samples could be treated within 48 h. After sampling, the samples were marked, sealed, and put into the cold storage device for inspection.

### Sample Collection

The samples were collected during the 180-days experiment, which was conducted in the CELSS platform located in Space Science and Technology Institute (Shenzhen), Shenzhen, Guangdong Province, China. The four crewmembers were quarantined for 180 days swabs. Sterile swabs were used for sampling from oropharynx and nasal cavity eight times. The swabs (155C conventional swab, Copan, Italy; ethylene oxide sterilization) were held on the handle and gently inserted into the oropharynx or nasal cavity, gently rotated 3–5 times, and then were removed slowly. Put the extracted samples into the sample collection tubes, break the handles, seal them and store them in the −80°C refrigerator to complete the sampling. Samples were taken 7 days before the confined experiment and 15, 30, 60, 90, 120, 150, and 180 days during the confined experiment. On a manned space station, in order to ensure that samples could truly reflect the growth of human microorganisms, on-orbit microbial samples were generally collected 1–2 days before the separation of the spacecraft and the space station (Novikova et al., 2006). So, nasal and oropharyngeal samples were collected on the same day or one day before delivery. Delivery modules happened once a month, and the samples were delivered from the capsule on that day. After sampling, the samples were marked, sealed, and stored in the cold storage device. The time between sampling and detection should not exceed 48 h. Then, DNA was extracted from samples, and microbiota analysis was carried out by using the molecular biological and genomics method.

### Illumina MiSeq Amplicon Sequencing

#### DNA Extraction

DNA was extracted with QIAGEN PowerSoil DNA Isolation Kit. DNA quality was determined by 1% agarose gel electrophoresis. The concentration of the extracted DNA was detected and adjusted. The DNA working solution was stored at 4°C, and the storage solution was stored at −20°C.

#### 16S rRNA Gene Amplicons for Sequencing

PCR amplification was performed on the V3–V4 regions of the 16S rRNA gene of samples. The PCR reaction (30 μl) was in triplets, including 22.4 μl ultra-pure water and 6 μl Taq&Go™ Mastermix (Biomedmix, Heidelburg MP, Germany), a forward and reverse primer of 0.3 μl, respectively (10 μM), and a 1-μl extracted DNA template. Amplification was performed in 35 cycles on the PCR instrument, which was set as follows: 95°C 45 s, 55°C, 45 s, 72°C 90 s, including initial denaturation at 95°C for 5 min and final elongation at 72°C for 10 min. Gel recovery and purification: the target strip was recovered by cutting, and purified samples were obtained. Quantification of each sample: a Qubit fluorescence quantitative analyzer was used to quantify each sample. Illumina TruSeq DNA Sample Preparation Guide was used to construct DNA library. Illumina MiSeq PE300 was used for sequencing.

### Sequencing Analysis

Sequence reads were analyzed with QIIME 1.9.1 (Caporaso et al., 2010) according to tutorials provided by the QIIME developers. After quality checking with Fastqc, the reads were assigned to each sample according to barcodes. Reads were merged to tags, and they are clustered into OTUs at 97% similarity. Based on OTUs and annotation results, composition difference analysis and microbial diversity were analyzed.

### Statistical Analysis

The comparisons of the microbial diversity were performed using R software (version 3.6.3). The Kruskal–Wallis test was used to compare the mean values of different groups, and *p* < 0.05 was considered statistically significant. The vegan package of R software was used to make principal coordinate analysis and draw the PCoA analysis chart.

## Results

**Figure 1:**
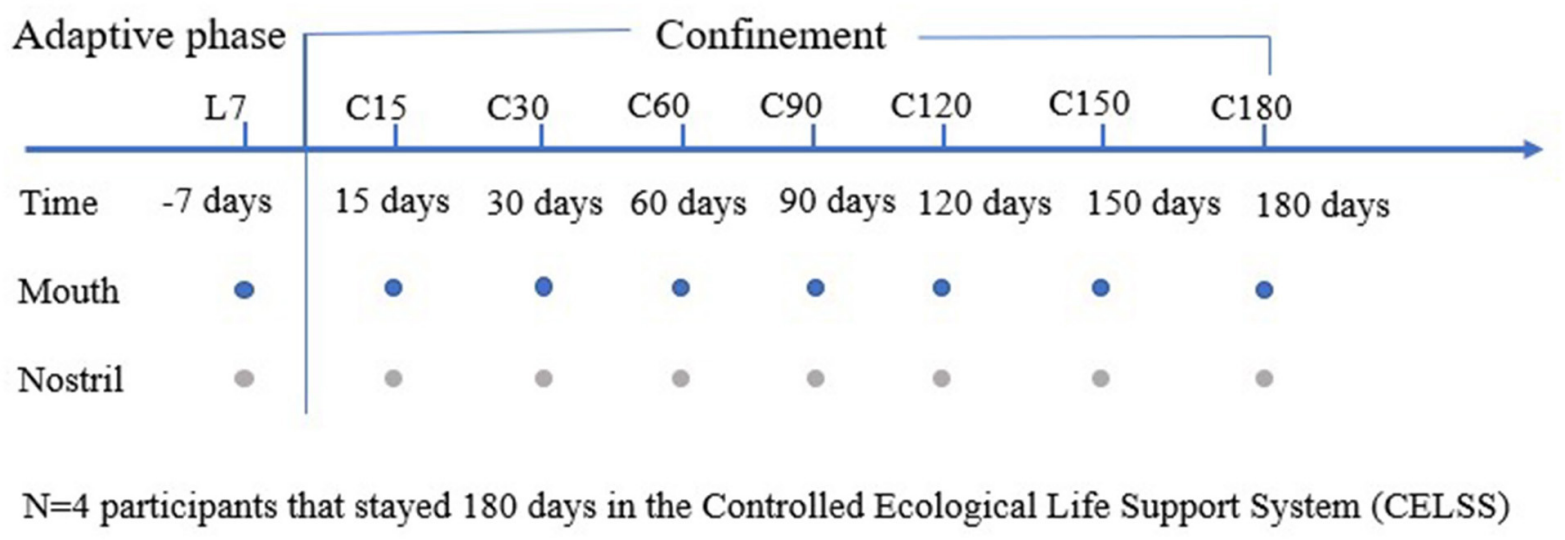
Schematic diagram of research design. The sampling time point is shown at the top. The numbers represent the number of days before (L-) and during (C-) confinement, and−7 days represents the 7th day before the confinement experiment. The colored circles represent the time points that were used to collect the sample specified on the left side of the figure.

The 64 swab samples were collected from the oropharynx and nasal cavity of the four crew members during isolation in the CELSS at eight time points over a 180-days period. The implementation of the whole experiment conforms to the design of the experiment (Yuan et al., 2019). The schematic diagram of the research experiment design is shown in Figure 1. The outline of the CELSS and the living and working places of the volunteers are shown in Supplementary Figure 1.

### Microbial Diversity Analysis of Nasal and Oropharyngeal Samples Under Confined Conditions

A total of 1,050,859 sequence readings were obtained from the nasal sample, and the median number of reads in the nasal sample was 31,266. A total of 666,675 sequence readings were obtained from oropharyngeal samples, and the median number of reads in oropharyngeal samples was 18,830.

The overall structure of microbial community composition was characterized by principal coordinate analysis (PCoA) of diversity differences. The results showed that the largest factor affecting the differences between samples was the sampling site (Supplementary Figure 2A). The microbial composition in the nasal cavity and oropharynx was distinctly different at the taxon of phylum (Supplementary Figure 2B). The nasal bacteria mainly belonged to Actinobacteria, Firmicutes, Proteobacteria, and Bacteroidetes, while Fusobacterial occupied a relatively high proportion in oropharyngeal microbiota in addition to the above-mentioned phyla. The microbiota of the same site had an individual difference in relative numbers (Supplementary Figures 2D,E). The index Chao of the oropharyngeal microbial community was higher than the nasal cavity, and it fluctuated more wildly in the nasal microbial community, but there was no statistical difference (*P* = 0.63) (Supplementary Figure 2C).

### Temporal Characteristics of Nasal and Oropharyngeal Microbiota Under Confined Conditions

**Figure 2:**
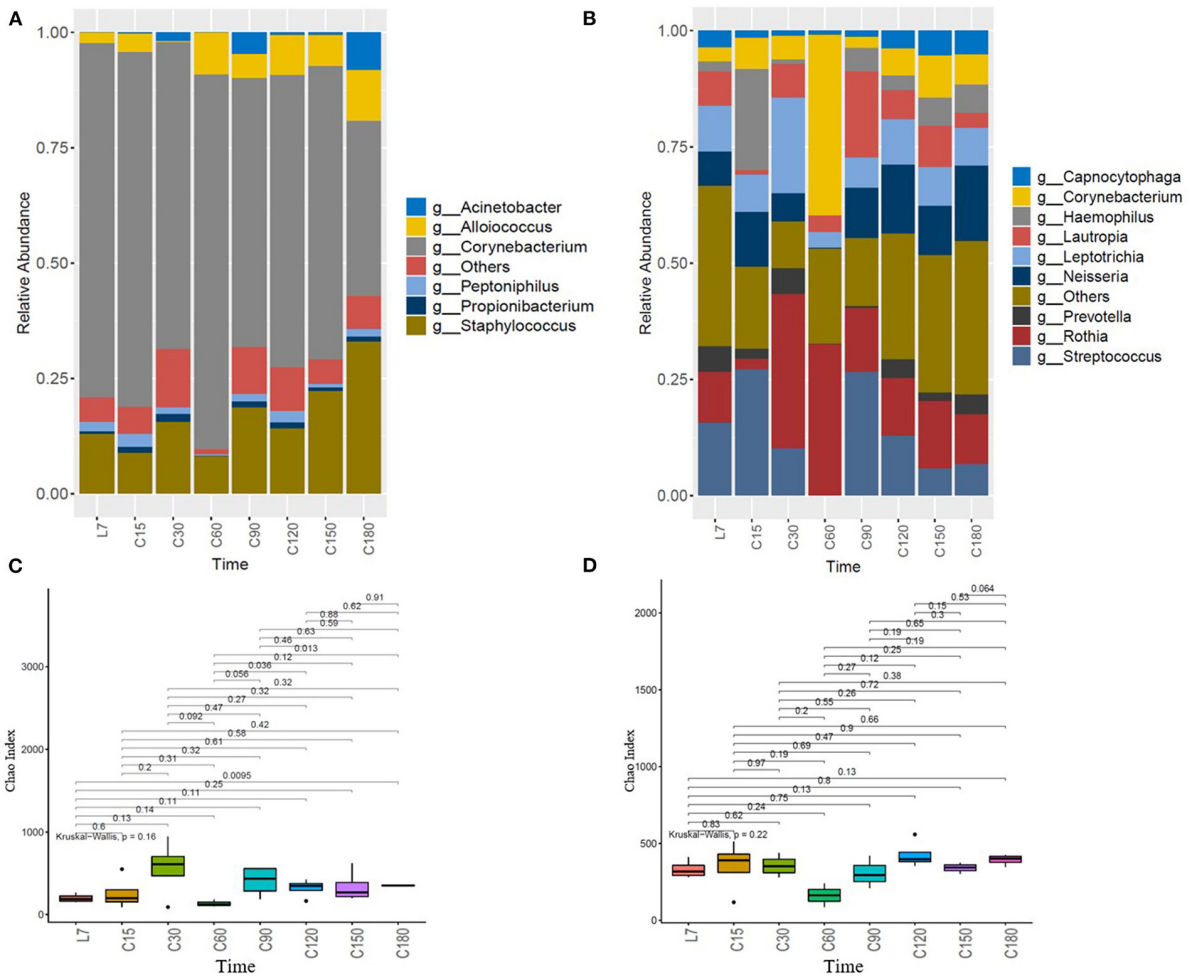
Dynamic analysis of the changes in the structure of microbiota over time. **(A)** Temporal characteristic of nasal microbiota, **(B)** temporal characteristic of oropharyngeal microbiota, **(C)** changes of nasal microbial index Chao with time, and **(D)** changes of oropharyngeal microbial index Chao with time. The horizontal axis represents the time point. L7 represents 7 days before the confined experiment, and C15, C30, C60, C90, C120, C150, and C180, respectively represent 15, 30, 60, 90, 120, 150, and 180 days during the confined experiment. The vertical axis represents the relative abundance of the genera.

QIIME 1.9.1 was used to generate the species abundance distribution diagram of multiple samples. According to the classification results, bacteria in the top six positions of richness detected in the nose samples mainly belong to *Corynebacterium, Staphylococcus, Alloiococcus, Peptoniphilus, Propionibacterium*, and *Acinetobacter*. Analysis results of nasal bacterial composition (Figure 2A, Supplementary Figure 3) showed that *Corynebacterium* and *Staphylococcus* were dominant, and with the extension of confined time, the relative abundance of *Corynebacterium* showed a downward trend (*P* = 0.083), and *Staphylococcus* and *Alloiococcus* increased (*P* = 0.48, *P* = 0.92).

The analysis results of the oropharyngeal microbial composition showed that (Figure 2B, Supplementary Figure 3) the dominant bacteria in oropharyngeal samples belonged to *Corynebacterium, Streptococcus, Neisseria, Rothia, Prevotella, Leptotrichia, Haemophilus, Capnocytophaga*, etc. The evenness of oropharyngeal dominant bacteria was higher than that of nasal dominant bacteria. On the whole, *Prevotella* showed a decreasing trend (*P* = 0.18), *Neisseria* showed an increasing trend (*P* = 0.34), *Capnocytophaga* decreased at first and then increased, while *Leptotrichia* showed little change.

The index Chao showed no obvious trend (Figures 2C,D), but it fluctuated greatly over time, especially at the time points of C30, C60, and C90. The index was relatively stable at the beginning and end of confinement.

It is worth mentioning that at the time point of C60, *Corynebacterium* has increased in the nasal cavity and oropharynx synchronously, the index Chao reduced and *Staphylococcus* in the nasal cavity and *Streptococcus* in oropharynx reduced.

### Dynamic Analysis of Individual Bacterial Community Structure During Confinement Time

There is some microbial specificity between individuals, so we analyzed the changes of individual bacterial community over time (Supplementary Figure 4). With the extension of the isolation time, as a whole, the nasal microbiota showed a decreasing trend of *Corynebacterium* (*P* = 0.083) and an increasing trend of *Staphylococcus* (*P* = 0.48), which was very obvious in volunteer 3 and 4. It should be noted that the abundance of *Alloiococcus* was higher and increased in the nasal cavity of volunteer 1, but it was rare in other volunteers. *Acinetobacter* was characterized abnormally high periodically in the nasal cavity of volunteer 4. *Propionibacterium* occupied a relatively low proportion in these dominant bacteria. *Staphylococcus* was very rarely in the nasal bacterial community of volunteers 2 and 4 before the confined experiment, while it was higher in the nasal bacterial community of volunteers 1 and 3. An interesting higher proportion of *Staphylococcus* was detected in the nasal bacterial community of all the volunteers during the confined experiment. The DNA extraction of nasal samples from volunteer 2 on day 180 and volunteer 4 on day 60 failed.

*Neisseria* in oropharyngiae (except occupant 1) showed an increased trend in the study (Supplementary Figure 5). An outbreak of *Rossella* happened 1–2 months after isolation and then returned to normal level. DNA was failed to be extracted from the oropharyngeal samples of volunteer 1 at day 60 and 90 and volunteer 4 at day 60.

**Figure 3:**
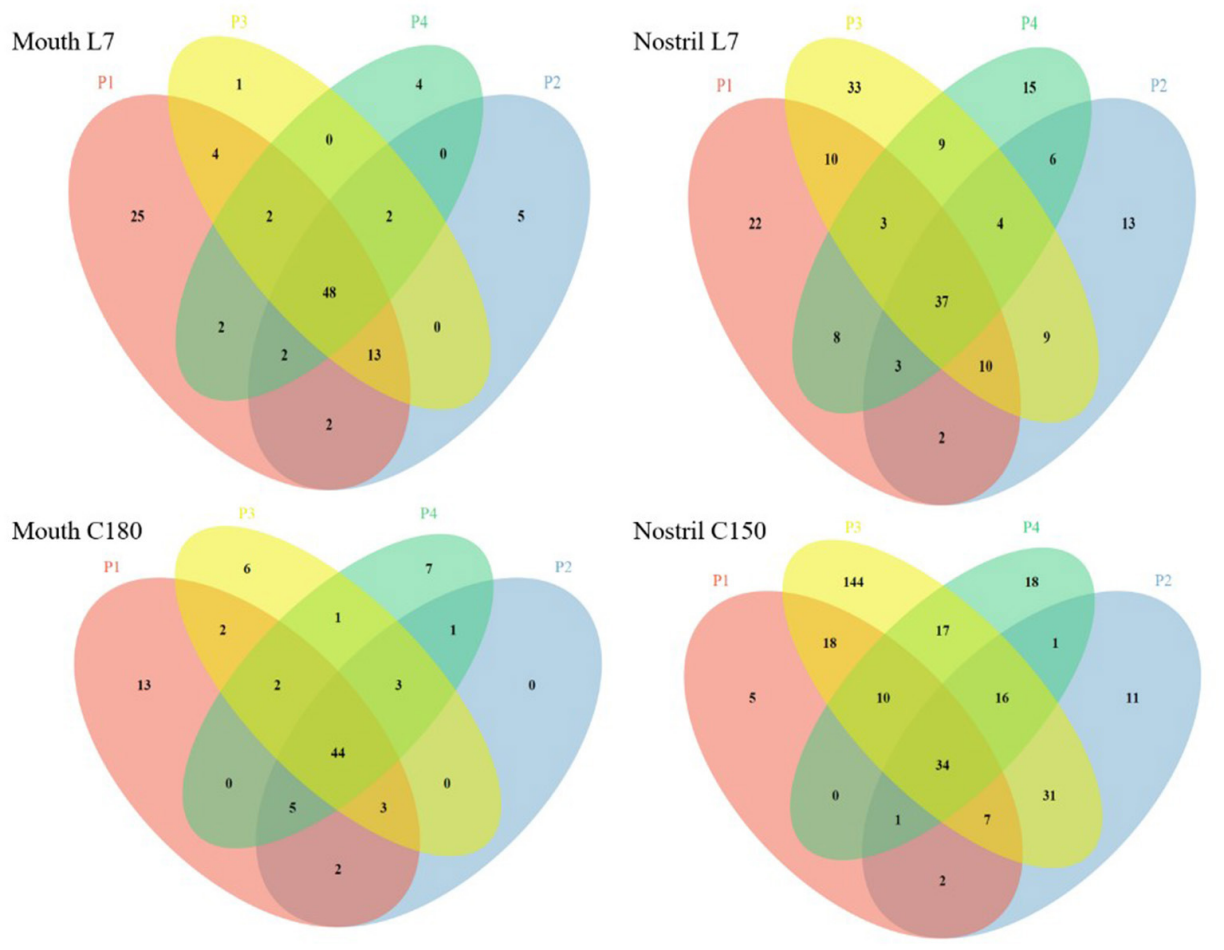
Venn diagram of oropharyngeal and nasal samples before and after confinement. Mouth L7 and Mouth C180 represent oropharyngeal samples taken 7 days before and 180 days after confinement, and Nostril L7 and Nostril C150 represent nasal samples taken 7 days before and 150 days after confinement, respectively. P1, P2, P3, and P4 are the numbers of four volunteers. Circles of different colors are used to represent different individual samples, and the numbers of overlapping areas represent the numbers of common OTUs among samples. The common OUT numbers increase after confinement in Nostril samples Nostril L7 and Nostril C150.

With 97% similarity, the OTU number of each sample was obtained. The Venn diagram was used to show the number of common and unique OTU numbers of multiple samples, and the OTU overlap among samples was visually displayed to reflect the microbial crossover between samples. We selected two time points of pre-confinement (L7) and confinement time 180 days (C180) for the Venn diagram analysis. Since it was failed to extract the DNA of nasal sample C180 from Volunteer 2, we chosed the nasal sample of confinement time 150 days (C150) instead of C180 for analysis (Figure 3). The number of common and unique OTU numbers of oropharyngeal samples was consistent before and after confinement. The common OTU numbers of nasal samples increased after confinement, which indicated that the nasal microbiota had the dynamic characteristic of evolving in the same direction after confinement, and the reason may be that airtight environmental factors caused the change of microbiota, while some microorganisms in the nasal cavity spread through the air and transfered between individuals in an airtight space.

### Distribution of Opportunistic Pathogens Before and After Confinement

Some genera of the dominant bacteria in the oropharyngeal and nasal microbial community contain pathogenic or opportunistic pathogens. According to the literature reports (Chonmaitree et al., 2017; González-Andrade et al., 2017; Katsoulis et al., 2019; Morinaga et al., 2019), several genera were selected from the dominant bacteria, which contained opportunistic pathogens. For example, *Staphylococcus* contains *staphylococcus aureus*, which produces enterotoxins that are harmful to health. *Neisseria* contains *N. meningitidis* which is a pathogenic bacterium of epidemic cerebrospinal meningitis, and it is mainly transmitted through the respiratory tract. *S. pyogenes* attached to *Streptococcus* can cause a variety of suppurative inflammation and hypersensitivity diseases, and *S. pneumoniae* can cause respiratory infections in human beings. The *A. otitis* attached to *Alloiococcus* is a pathogenic factor in adult secretome otitis media. *P. acnes* in *Propionibacterium* can cause skin inflammation and is the pathogen of acne.

**Figure 4:**
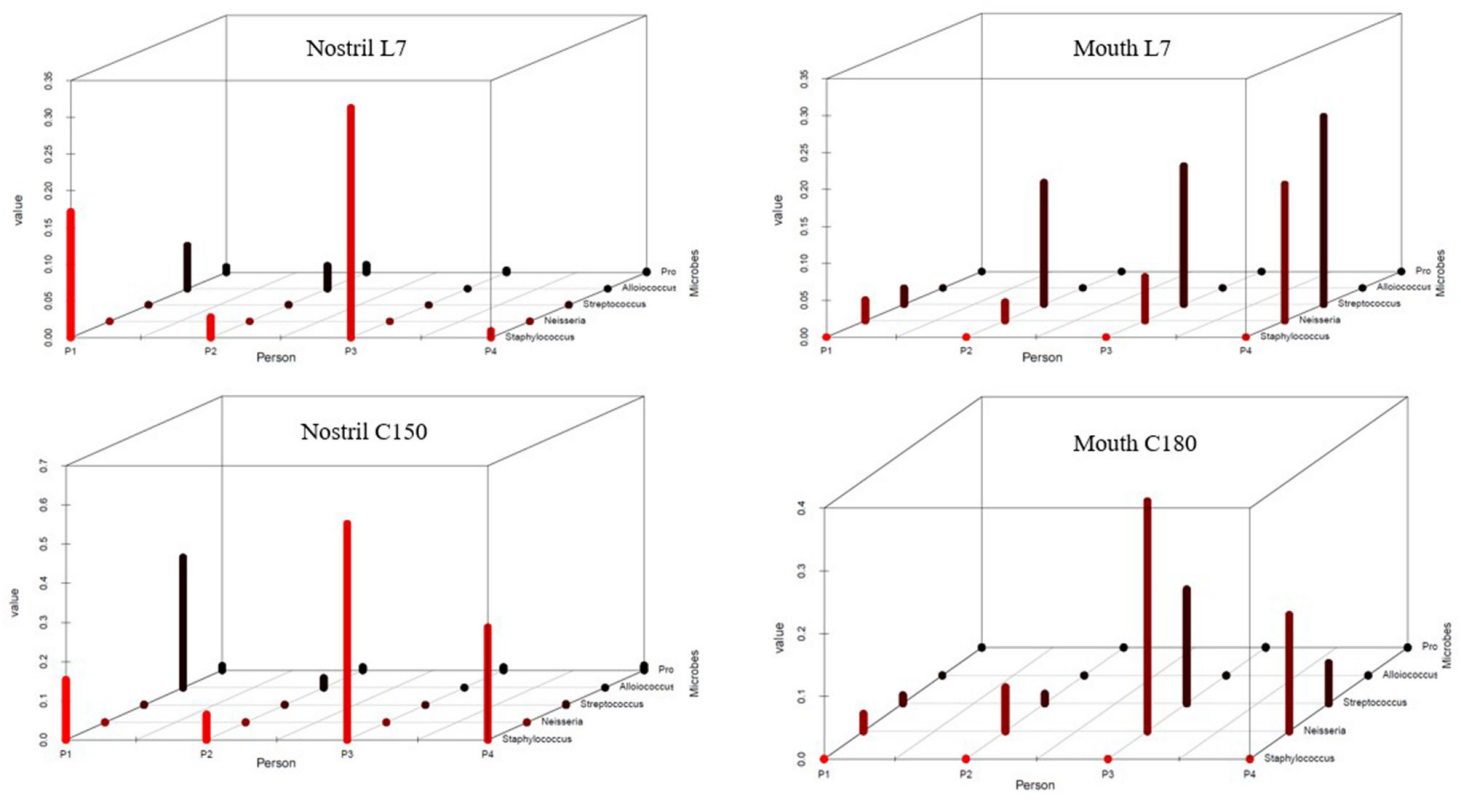
Changes in the content of potential pathogenic bacteria before and after confinement. The bacterial distribution 7 days before confinement (L7) and 180 days after confinement (C180) in different individuals was statistically analyzed. The horizontal axis P1, P2, P3, and P4 represented volunteers, the vertical axis represented the content value of bacteria, and the oblique axis represents the names of the bacterial genera. Nostril L7 and Nostril C180 represented the nasal samples taken 7 days before and 180 days after confinement. It was failed to extract in which the DNA of 180 days' nasal sample of Volunteer 2 and it was replaced by 150 days' nasal sample of Volunteer 2. Mouth L7 and Mouth C180 represented oropharyngeal samples taken 7 days before and 180 days after confinement, respectively. Note that the scale values used on the vertical axis were different and need to be distinguished.

To reveal the changes of the above opportunistic pathogens in the human body in an isolation environment, we tried to compare the difference of the genera containing pathogen bacteria before and after the isolation (Figure 4, Supplementary Table 2). It was worth mentioning that the sequencing sequence of the 16s rRNA gene V3–V4 area could not distinguish the species level; however, the genera containing potentially pathogenic bacteria that we had listed could reflect the increased risk of infection by opportunistic pathogens from the side. It was failed to extract DNA from the nasal sample of C180 of Volunteer 2, and C150 was used for alternative analysis. The figure showed obvious increase in *Staphylococcus* of the nasal cavity and in *Neisseria* of the oropharynx after confinement, suggesting an increased risk of infection.

## Discussion

In space, the special environmental factors may change bacterial community structure in the human body of astronauts, and break the microbiota balance. Working on-orbit results in decreased immunity and increases the risk of pathogenic infections. These infections may occur in the respiratory tract, the digestive tract, urinary tract, and skin, affecting the individual health and subjective experience. At the same time, these could affect other individuals, even the whole crew, through the migration of bacteria. Therefore, the study of the human bacterial community structure in the confined environment is of great significance for the prevention of infectious diseases during long-term flight on-orbit in the future. In our study, the temporal characteristics of the oropharyngeal and nasal microbiota structure in crewmembers were researched, in order to provide reference for revealing possible effects of microbiota balance on respiratory health in a closed environment.

The culture method is the most commonly used method for studying microbes on the space station, and although there have been attempts to sequence them on-orbit, conditions on the space station have limited the use of sequencing technology on-orbit. Currently, on-orbit microbial samples are returned to the earth for high-throughput sequencing and analysis for microbiota studies on space station. However, due to the limitation of return time, dynamic analysis of microbiota cannot be carried out immediately, and long-term preservation and space transportation may lead to the risk of analysis error. Therefore, many scholars simulate the space environment on the ground to dynamically study the changes of environment and human microbiota. In order to monitor the microbial characteristics of the closed system on the ground in real time, the researchers designed different experiments and simulated buildings to conduct research. Russia conducted a confined Mars500 habitat, and simulated flight and landing on Mars, using high-throughput sequencing technology to study the dynamic changes of the intestinal microbiota structure over time in six volunteers (Schwendner et al., 2017). Then, in the expansion of the lunar/Mars habitat simulation test was carried out, high-throughput sequencing technology was used to survey bacterial community in the air, and the results showed that in closed habitats, bacterial structure of air had a close relationship with human existence (Mayer et al., 2016).

The CELSS system is an excellent platform to simulate the closed environment of the space capsule on the ground. The confined experiment of four people for 180 days provided an opportunity to dynamically track the changes of the crews' own microbiota and the microbiota of others living in close proximity for a long time. During the 180-days confinement simulation experiment, human respiratory tract microbial samples were collected at eight time points, and the microbiota were continuously observed over time. This is the first time to study the changes of respiratory microbiota over time through CELSS.

This study aimed to reveal the effect of long-term confinement on the composition and diversity of nasal and oropharyngeal microbiota. We found that nasal cavity and oropharynx were independent in terms of microbiota structure and diversity (Supplementary Figures 2A,B), and this independence did not disappear with the extension of confinement time, indicating that the physiological environment and microecological environment of the growth site of the microbiota were the most important influential factors on the microbiota. Studies had investigated the temporal stability and diversity of skin microbiota, regardless of the sampling interval, and despite the skin community's constant exposure to external factors, the stability of the microbiota still existed, while the nature and degree of this stability was highly individualized (Oh et al., 2014).

In terms of bacterial composition, the nasal bacteria mainly belong to Actinobacteria, Firmicutes, Proteobacteria, Bacteroidetes, etc. In addition to the abovementioned phyla, the Fusobacterial occupies a relatively high proportion in oropharyngeal bacteria. At the level of genus, the dominant bacteria of nasal cavity are *Corynebacterium, Staphylococcus, Diaphylococcus, Propionibacterium*, etc., while the dominant bacteria of oropharynx are *Corynebacterium, Streptococcus, Neisseria*, and *Prevotella*. Previous studies have shown that the main members of the microbial ecosystem in the nasal cavity are usually Actinobacteria (containing *Corynebacterium* and *Propionibacterium*) and Firmicutes (*Streptococcus* in children and *Staphylococcus* in adults), while the abundance of anaerobic bacteria in Bacteroidetes is low (Camarinha-Silva et al., 2014; Oh et al., 2014). At the generic level, *Corynebacterium, Propionibacterium*, and *Staphylococcus* are the most common genera in the nasal cavity (Zhou et al., 2014). Previous studies have shown that bacterial biodiversity and uniformity vary greatly in the upper respiratory tract; thus, the oropharynx and oral cavity have the highest biodiversity and uniformity (Charlson et al., 2010; Zhou et al., 2013). On the contrary, similar to other areas covered by human skin epithelium, the nasal cavity shows low biodiversity (Grice and Segre, 2011).

During 30–90 days of confinement, the structure of nasal and oropharyngeal microbiota changed (Figures 2A,B), and the index Chao of the microbiota fluctuated greatly (Figures 2C,D). The confinement environment disturbed the nasal and oropharyngeal microbiota, especially after 30 days of confinement. In addition, the oropharyngeal microbial diversity was higher than the nasal cavity, and the index Chao of nasal cavity fluctuated more, but there was no statistical difference (*P* = 0.63) (Supplementary Figure 2C). The results of behavioral monitoring and psychological state assessment in this project showed that after 1 month of detention, behavioral flow reflecting global activity decreased 1.5- to 2-fold. Psychological questionnaires revealed a decrease in hostility and negative emotions but an increase in emotional adaptation suggesting boredom and monotony (Biesbroek et al., 2014). Physiological and psychological changes in volunteers in the isolation environment may trigger a series of physiological stress changes, such as immunity and secretion of respiratory antibacterial substances, which may affect the respiratory microbiota. According to NASA's research data, the exchange or migration of pathogenic microorganisms between passenger groups occurred in flight missions over 18 days (Crucian et al., 2016a). This is another explanation for the change in the microbiota after 30 days of confinement.

Dynamic analysis of the microbiota structure over time found that *Staphylococcus* and *Alloiococcus* showed an upward trend, while *Corynebacterium* showed a downward trend (Figure 2A). *Neisseria* showed an upward trend in oropharyngeal microbiota, while *Corynebacterium* showed a large fluctuation (Figure 2B). Early nasopharyngeal microbial studies showed that high abundance ratios of *Corynebacterium* and *Moraxella* marked a more stable structure, and poor stability was characterized by high abundance of *Streptococcus* and acquired *H. influenzae*, and the microbiota characteristics were correlative with reported higher incidence of respiratory tract infections and asthma in the early stages of life (Stubbendieck et al., 2019). Microbial communities characterized by high abundance of *Corynebacterium* and lack of the presence of *H. influenzae* and *S. pneumoniae*, suggesting colonization resistance to these potential pathogens. In this study, the abundance of *Corynebacterium* was the highest, indicating that the structure of the bacterial community was stable. However, during the confined period, *Corynebacterium* showed a downward trend, while *Staphylococcus* showed an increasing trend, suggesting that confinement had an adverse effect on the structural stability of the nasal bacterial community.

Worth noting is that although *Corynebacterium* showed a trend of decrease in the nasal cavity, but at the C60 time point, there was a dramatic rise of *Corynebacterium* in nasal and oropharyngeal microbiota collaboratively (Figures 2A,B), together with much lower index Chao (Figures 2C,D), at the same time *Staphylococcus* in the nasal cavity and *Streptococcus* in oropharynx greatly reduced. Studies have shown that *Corynebacterium* competes for limited iron resources in the nasal cavity by producing the iron-chelating vector, which is related to the ability to inhibit *Staphylococcus*. At the same time, the lipase secreted by *Corynebacterium* can lyse the triacylglycerol in the nasal cavity and produce the free fatty acid which is resistant to microorganisms, thus inhibiting the growth of *Streptococcus in vitro* (Marik, 2001).

Oropharynx connects the mouth, nasopharynx, larynx, lower respiratory tract, and gastrointestinal tract and it is exposed to exogenous and endogenous microorganisms. Thus, the species pool contained in oropharyngeal microecology is usually large and a highly diverse of bacterial community can be observed in adults. The oropharynx is also the niche of potentially pathogenic bacteria that may cause local (pharyngitis) or diffuse (lung) diseases (Mermel, 2013). *Neisseria* contains *N. meningitidis* which is a pathogenic bacterium of epidemic cerebrospinal meningitis, and it is mainly transmitted through the respiratory tract. *Rothia* is a class of symbiotic bacteria that is widespread in the mouth. In this study, *Neisseria* in the oropharynx (except volunteer 1) showed an increasing trend. An outbreak of *Rossella* happened 1–2 months after confinement, then it returned to normal (Supplementary Figure 5). Disturbance of the microbiota may lead to increased risk of infection. The analysis suggests that it may be related to the inadaptability to the confined environment at the initial stage of experiment, the insufficient proficiency of each item, the tension of the volunteers, and the great physical and mental pressure.

We respectively analyzed the changes in the microbiota of four volunteers over time and compared them crosswise. Nasal *Staphylococcus* in volunteers 2 and 4 makes up only a very small percentage, while in volunteers 1 and 3 they makes up a bigger percentage before confined experiment. Nasal *Staphylococcus* accounted for a bigger percentage in all volunteers after 180 days confined experiment (Supplementary Figure 4). To explore this phenomenon, we used the Venn diagram to show the common and unique OTU numbers of samples before and after confinement, and there is a tendency of convergence in the nasal cavity (Figure 3), so we speculated that four volunteers had a long and close contact in a confined space, resulting in a microbial transfer between them. The microorganisms carried on the surface of the volunteers are in direct contact with the environment and can have an impact on the environment and then can be transferred. According to NASA's research data, the exchange or migration of pathogenic microorganisms between the crew takes place in a flight mission of more than 18 days (Crucian et al., 2016a). The understanding of the population status of the nasal cavity and oropharyngeal microorganisms is beneficial to the prediction and prevention of the occurrence of on-orbit infectious diseases. Studies have shown that the microbiota on the internal surface of the International Space Station is similar to the microbiota on the crew's skin. These data provide reference for future microbial monitoring work of the ISS and crew and the control of microbial contamination of manned spacecraft (Avila-Herrera et al., 2020).

To reveal the changes of pathogenic bacteria over time during confinement, considering our sequencing method could not accurately locate the level of species, so we chose genera for symbolic judgment (Figure 4). It could be seen that *Staphylococcus* in the nasal cavity and *Neisseria* in the oropharynx increases and spread with the extension of the confinement time. Venn diagrams also reflected a similar situation (Figure 3), and common OTU numbers of nasal samples increased after confinement. Nasal microbiota had the same direction of evolutionary dynamic characteristic, and the reason may be that closed environmental factors caused the change of bacterial community; some nasal microorganisms in airtight space spread through the air and transferred between individuals.

The results of the bacterial census of the International Space Station showed that *Staphylococcus, Bacillus*, and *Corynebacterium* were among the top three in the detection rate of environmental microbial samples from International Space Station (Venkateswaran et al., 2014). *Staphylococcus* are a bacteria of human origin, and it is also pathogen that induce infectious diseases. *Staphylococcus aureus* are a conditional pathogenic bacterium that NASA attaches great importance to, and crew need medical treatment whose naval *S. aureus* is resistant to drugs in preflight medical examination. The purpose is to reduce infectious diseases caused by migration and exchange of conditional pathogenic bacteria between susceptible people (individual difference) during long-term living closely (Ramakrishnan et al., 2013). A large number of studies have found that chronic nasosinusitis in the upper respiratory tract of patients is associated with higher abundance of *Staphylococcus* accompanied by decreased bacterial diversity (Feazel et al., 2012; Jervis Bardy and Psaltis, 2016; Muluk et al., 2018), and *Staphylococcus* is also positively correlated with other respiratory diseases (such as allergic rhinitis and asthma) (Voorhies et al., 2019). Although other factors experienced by ISS crew members may play a role in the development of upper respiratory symptoms, the increased relative abundance of *Staphylococcus* in the nasal bacterial community is consistent with these symptoms (Crucian et al., 2016; Paetzold et al., 2019).

The bacterial index Chao did not show an obvious trend (Figures 2C,D), but it fluctuated greatly over time, especially at the time points of C30, C60, and C90. The index Chao was relatively stable at the beginning of confinement and before confinement ended, showing a certain stability. Host state and complex interactions of the disturbance of environment may influence the local change of bacterial community, and some species could flow in some individuals and parts and influence the structure and function of the microbiota in the short term, while microbiota maintain certain stability in the long run. As with the study of skin microbiota, the most typical example is that the human use probiotics to improve skin health (Oh et al., 2016), but skin microbiota seem to have inherent stability (Crucian et al., 2016).

Two respiratory sites were detected in this study; in another article, we also discussed the changes of the skin microbiota (Bingmu et al., 2018). According to the on-orbit disease investigation and analysis, intestinal tract, urinary tract, and eye are also common sites of infectious disease, in addition to the respiratory disease, diarrhea, urinary tract infection with aeruginosa, and eye sty caused by *S. aureus* also happened on-orbit (Hurlbert et al., 2010; Hodkinson et al., 2017). These infectious diseases prompted us to analyze microbial microbiota of these parts and carry out astronaut individualized microbiota characteristic analysis, especially for long-term orbits. Some research directions such as real-time acquisition and detect microbial samples observe the change of environmental and the astronaut's microbiota, prevent infectious disease, also have important research value for future long-term orbit, and these works still need to continue to explore.

## Conclusion

In this study, 16S rDNA Illumina Miseq high-throughput sequencing was used to analyze the effects of nasal and oropharyngeal microbiota from crewmembers' long-term confined in the CELSS. The results showed that the structure of the nasal and oropharyngeal microbiota varied greatly, the individual bacterial community structure and the diversity changed with time. *Staphylococcus* in the nasal cavity increased and showed the characteristics of inter-individual transfer, suggesting that the microbiota structure and health of the respiratory tract could be affected by living in a closed environment for a long time.

The imbalance of the respiratory microbial community and the increase of opportunistic pathogens may be the inducement of respiratory diseases, but it does not mean that the increase of opportunistic pathogens will cause diseases; in addition, it is closely related to individual immune function. In the future, we will continue to carry out relevant studies on opportunistic pathogens. In the following work, we will also use a higher-resolution sequencing method to locate the species level and conduct in-depth analysis on pathogenic bacteria that may cause respiratory diseases.

## Data Availability Statement

The datasets presented in this article are not readily available because of the data confidentiality clause of the project sponsor. Requests to access the datasets should be directed to Space Science and Technology Institute (Shenzhen, http://www.szsisc.com/).

## Ethics Statement

The research program was approved by the Ethical Committee of the ACC (China Astronaut Reasearch and Training Center Beijing, China) and complied with all the guidelines in the Declaration of Helsinki. Each volunteer was informed of the content and schedule of the study and signed an informed consent form. Participants can withdraw from the study at any time.

## Author Contributions

YC and CX: experimental design. YC, BX, and CZ: methodology. ZL, ZC, and JL: investigation. YC and BX: writing—original draft. CX, QX, and ZL: writing—review and editing. BX and QX: funding acquisition. All authors agree to be accountable for the content of the work.

## Conflict of Interest

The authors declare that the research was conducted in the absence of any commercial or financial relationships that could be construed as a potential conflict of interest.
