# Supplementary figures and images for: Temporal Characteristics of the Oropharyngeal and Nasal Microbiota Structure in Crewmembers Stayed 180 Days in the Controlled Ecological Life Support System
Source: Front Microbiol. 2021 Feb 3;11:617696. doi: 10.3389/fmicb.2020.617696 (PMC7886687; doi:10.3389/fmicb.2020.617696)

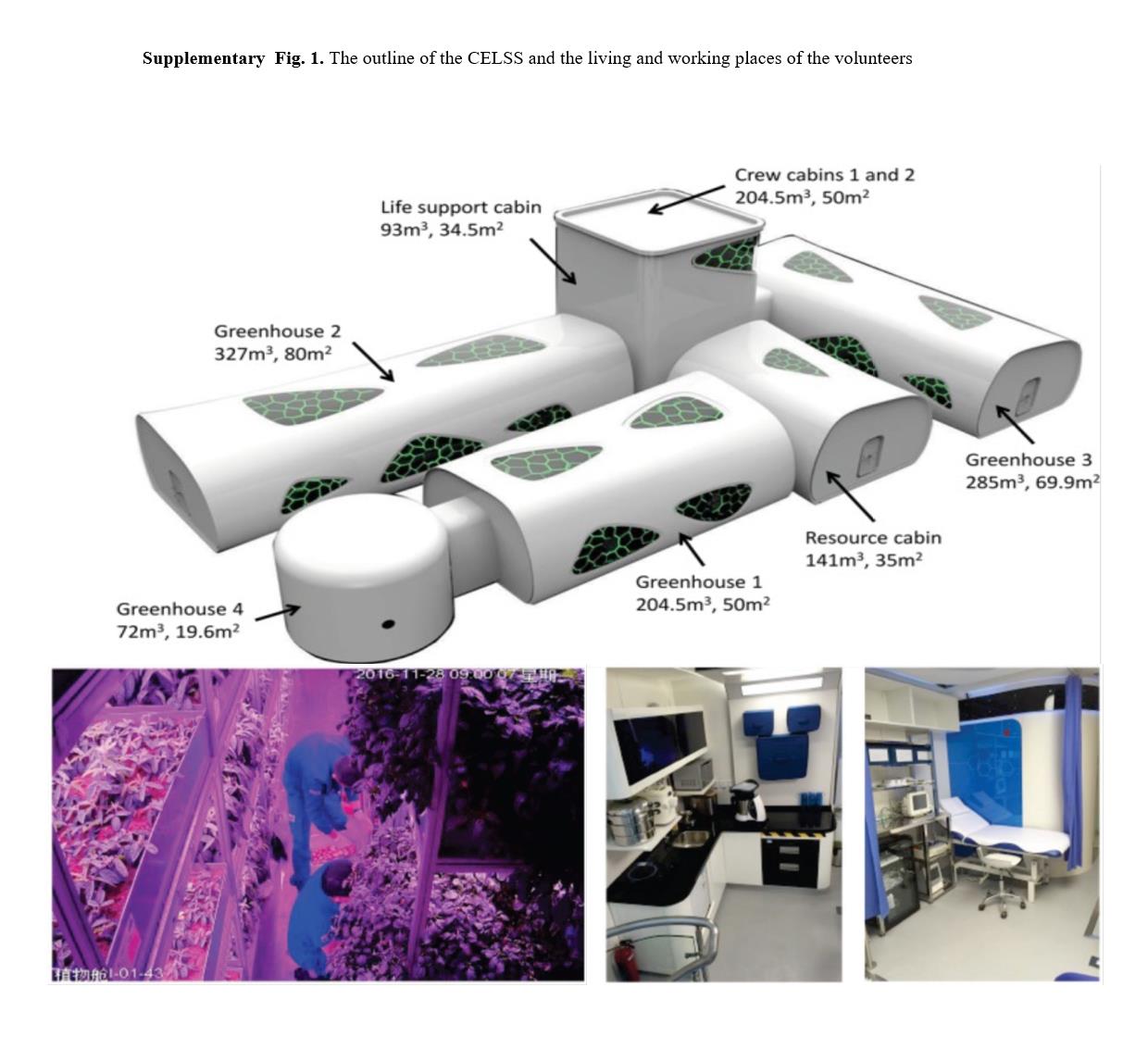

Supplement: Supplementary file 3 [file Image_1.JPEG]

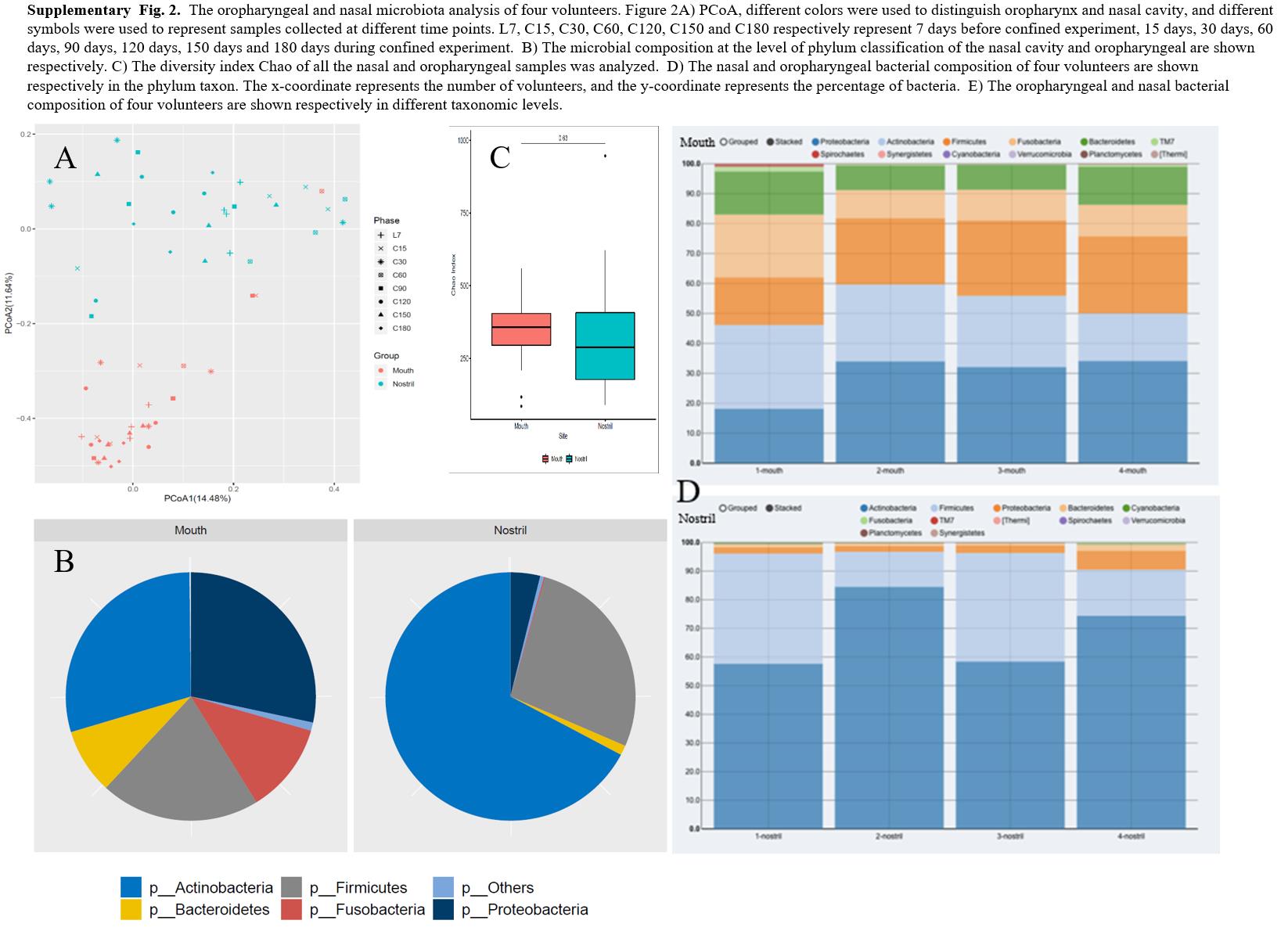

Supplement: Supplementary file 4 [file Image_2.JPEG]

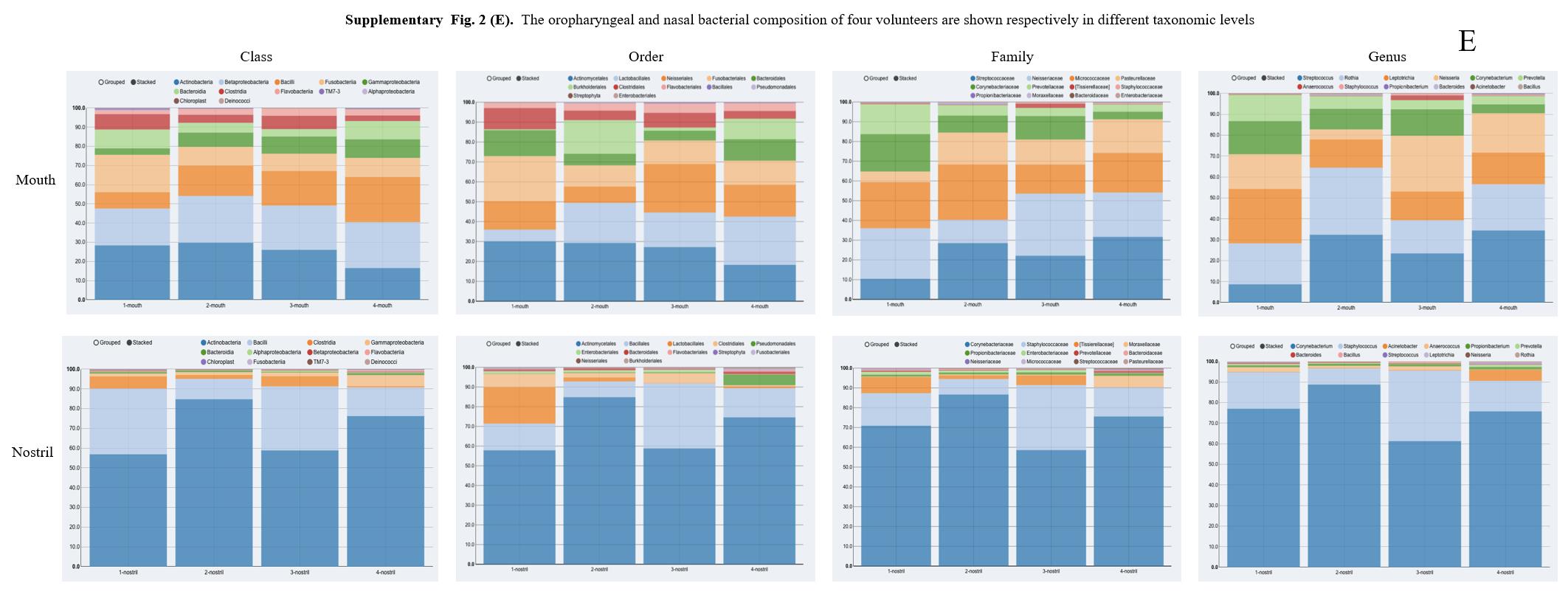

Supplement: Supplementary file 5 [file Image_3.JPEG]

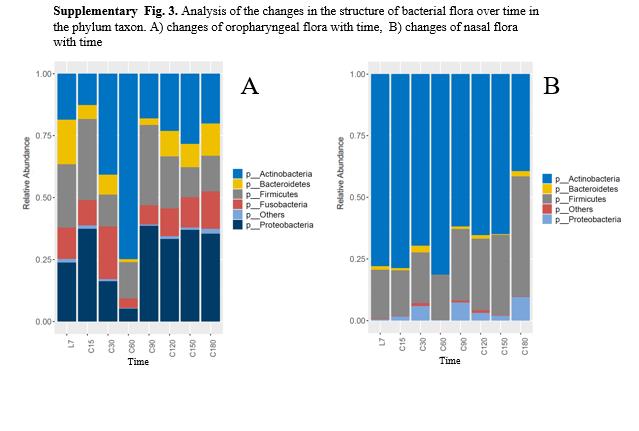

Supplement: Supplementary file 6 [file Image_4.JPEG]

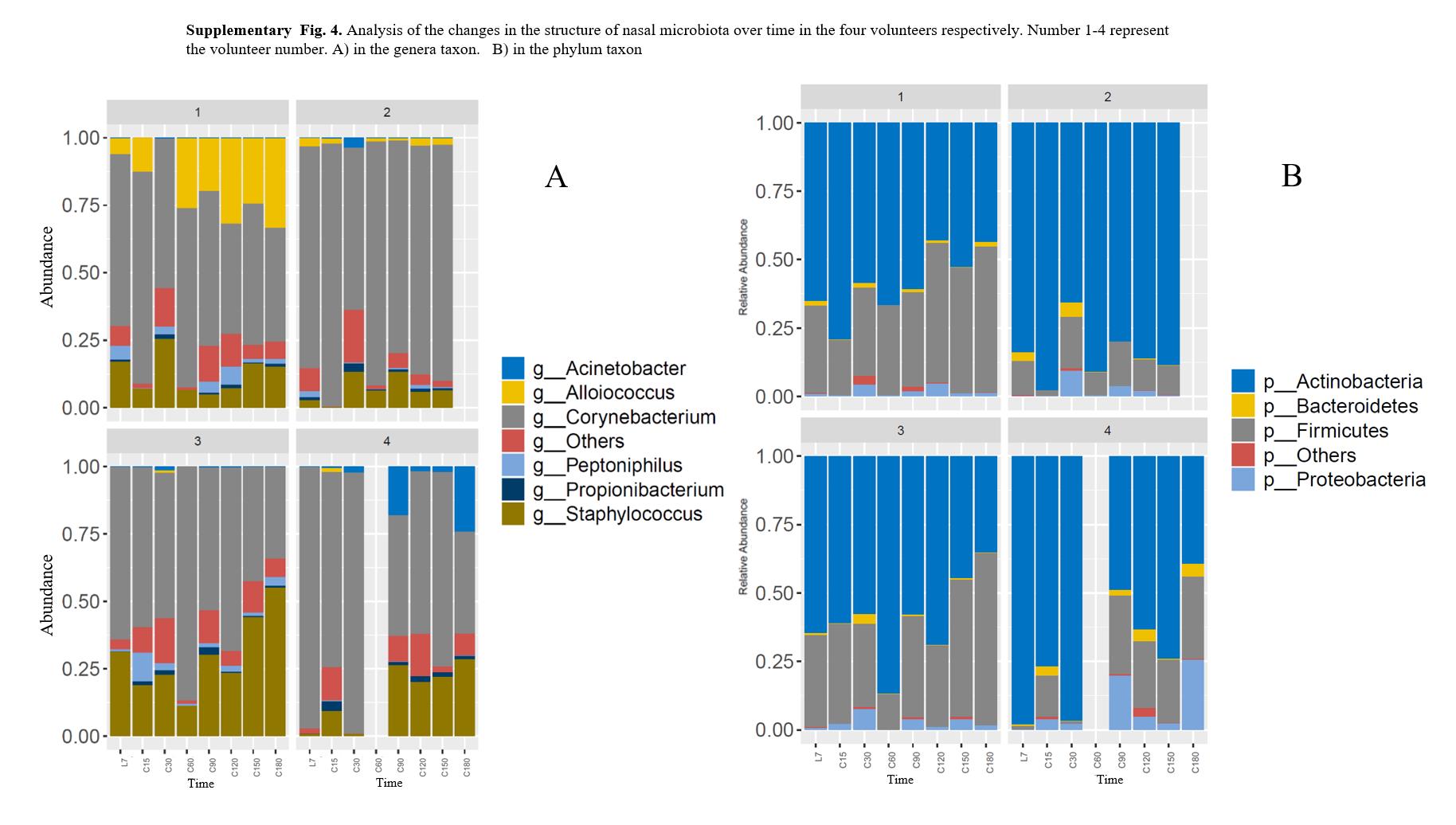

Supplement: Supplementary file 7 [file Image_5.JPEG]

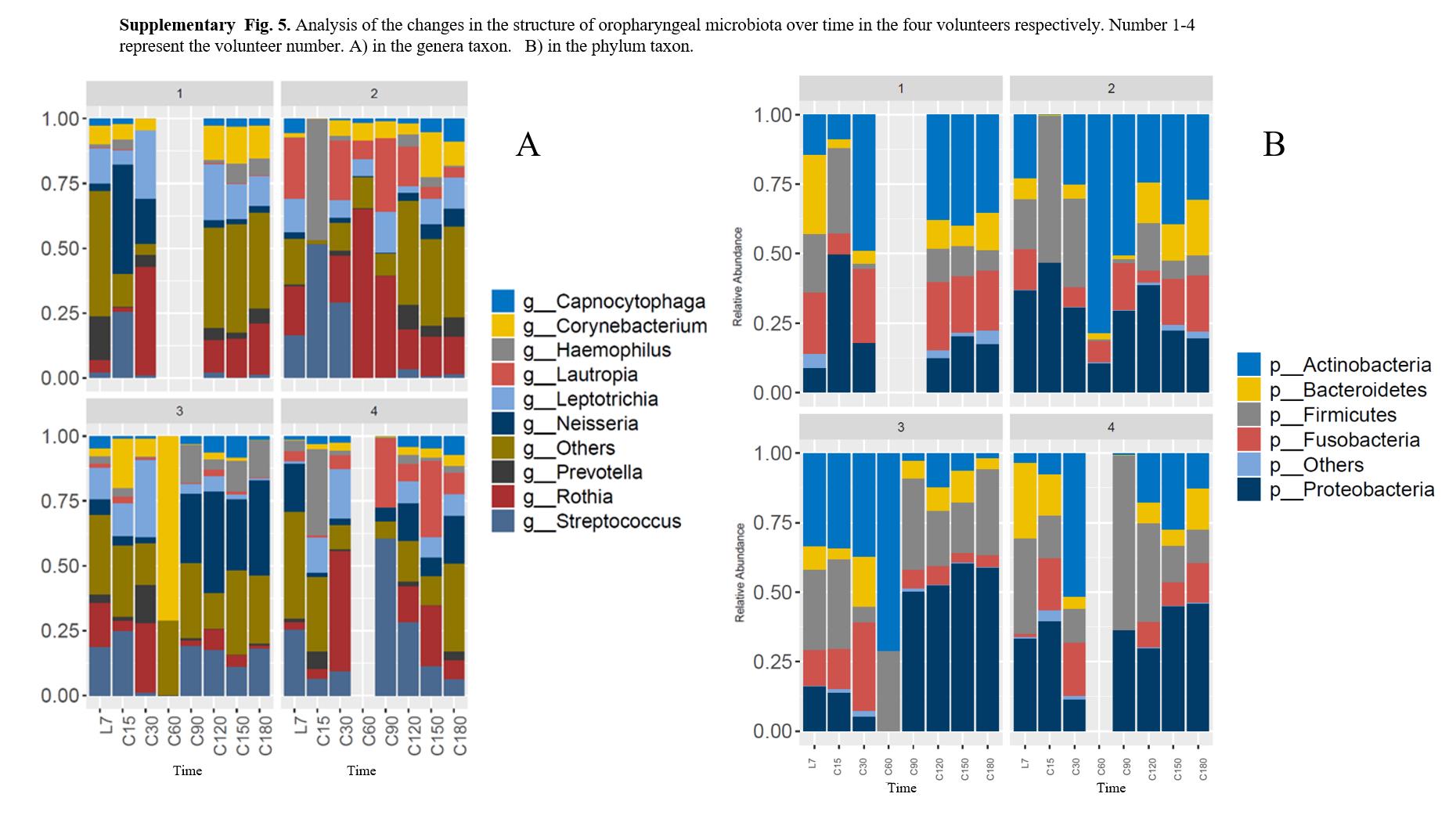

Supplement: Supplementary file 8 [file Image_6.JPEG]
